# Metabolic origins of childhood asthma

**DOI:** 10.1186/s40348-015-0017-3

**Published:** 2015-04-01

**Authors:** Hartmut Grasemann

**Affiliations:** Division of Respiratory Medicine, Department of Pediatrics, and Program in Physiology and Experimental Medicine, Research Institute, Hospital for Sick Children, and University of Toronto, 686 Bay St., 9th floor, Toronto, ON M5G 0A4 Canada

**Keywords:** Obesity, Diabetes, Metabolism, Asthma, Intrauterine programming

## Abstract

Childhood obesity and incidence of asthma are increasing globally. The parallel increase of the two suggests that obesity and asthma may be related and that abnormalities in the lipid and/or glucose metabolism may contribute to the pathogenesis of asthma. The clinical presentation of obese asthma is distinct from other asthma phenotypes and depending on age of onset of symptoms. Asthma in obese people tends to be more severe, not typically associated with allergy, and less responsive to standard anti-inflammatory therapy, including corticosteroids. Obesity and obesity-related comorbidities may lead to asthma via a number of mechanisms including changes in lung mechanics, the nitric oxide metabolism, and by causing inflammation. Furthermore, evidence suggests that nutrition during pregnancy contributes to intrauterine immune and metabolic programming in the offspring, which may have major influences on predisposition to cardiovascular, metabolic, and allergic diseases, including asthma, later in life. This review will highlight some suggested mechanistic links between obesity and diabetes with asthma.

## Introduction

This review will summarize reported evidence of a possible link between obesity, diabetes mellitus, and asthma highlighting the potential roles of inflammation and abnormalities in the L-arginine/nitric oxide metabolism as well as metabolic and immune programming. An association between a greater body mass index (BMI) and increased risk of asthma in both children and adults has been repeatedly shown in prospective, population-based studies [1-5]. Nearly 30% of children are overweight or obese in the United States [6], where childhood obesity rates have tripled in the past three decades [7,8]. It is well known that obesity is associated with the development of insulin resistance and type II diabetes [9], but the increase in obesity also parallels an increase in the incidence of allergic disease in early life, particularly food allergy [10,11], as well as asthma [12,13].

The simultaneous increment in the prevalence of these conditions among children could be incidental but may also suggest common underlying predisposing or contributing factors and/or a causal relationship of metabolic conditions with allergic disease and specifically asthma [14-18]. For instance, the International Study of Asthma and Allergies in Childhood (ISAAC) Phase Three study demonstrated significant associations between overweight/obesity with asthma and eczema, but not rhinoconjunctivitis [19]. Interestingly, in some studies, the association between obesity and asthma was found to be stronger in non-atopic than atopic children [20,21], and the association between BMI and asthma seems to be stronger among girls than boys [1,22-24]. Clinically important, obese asthmatics tend to present with more and severe symptoms and have poorly controlled asthma although they use more medications, and recovery from acute exacerbations is slower compared to their lean counterparts [25-28]. While it is conceivable that intensified treatments with high-dose inhaled or systemic corticosteroids as well as reduced physical activity in obese asthmatics contribute to weight gain, it is also important to notice that asthma-related outcomes in obese asthmatics can be improved with weight loss [29-31]. This not only provides further evidence for a causal role of obesity for asthma but also implies that weight loss should be attempted as supportive intervention in the treatment of obese/overweight asthmatics.

## Review

### Phenotypic distinction

A number of studies identified obesity in children and adolescents as a risk factor for asthma, more severe asthma, poor asthma control, frequent exacerbations, and asthma-related hospitalizations [17,25,26,32-34]. The age of the weight gain may play a role, as pronounced weight gain early in life was identified as a risk factor for developing asthma in the first 6 years of age [35]. Two types of asthma in obese subjects can be distinguished by age of onset and clinical presentation. Early-onset asthma in obese presents before the age of 12 years, has no gender preference, and is characterized by severely decreased pulmonary function, significant airway hyperresponsiveness, and poor asthma control. These patients are atopic; serum immunoglobulin E (IgE) is increased, airway inflammation is eosinophilic, and fraction of exhaled nitric oxide (FeNO) is high [28]. In contrast, obese late-onset asthmatics become symptomatic after the age of 12 and are predominantly females without atopic characteristics. Compared to early-onset asthmatics, they have little airway obstruction with less airway hyperresponsiveness and better asthma control. This phenotype has a TH2 low profile with predominant neutrophilic airway infiltration [36], which may at least in part explain the poor response to standard asthma therapy in this patient group.

### Effect of obesity on pulmonary function and airway responsiveness

Respiratory symptoms including shortness of breath with exercise in obese asthma may be mistaken for being related to the obesity alone; thus, pulmonary function testing should be performed to rule out a diagnosis of asthma. However, increased body fat deploys direct mechanical effects on the respiratory system independent of asthma. Obese individuals breathe at low tidal volumes and high respiratory rate. Increased BMI leads to a reduced functional residual capacity and expiratory reserve volume [15,16]. Pulmonary function tests in patients with BMI >30 may in addition reveal decreased total lung capacity, vital lung capacity, and residual volume [37]. Diabetes and insulin resistance have also been reported to be linked to lung function, and insulin resistance is associated with reduced lung function independent of diabetes, even after controlling for BMI [38-41].

Interestingly, impaired respiratory mechanics in obese people may also contribute to the development of asthma. Breathing at lower lung volumes results in airway narrowing and thereby increased resistance, which may contribute to airway hyperresponsiveness (AHR), a characteristic feature of asthma [42-45]. Whether obesity enhances or modifies AHR in children with asthma however remains unclear, as studies have revealed inconsistent results. No effect of BMI on AHR was found in participants of the Childhood Asthma Management Program (CAMP) after adjusting for baseline forced expiratory volume in the first second (FEV_1_); in the CAMP study, more than 1,000 children with mild to moderate asthma were enrolled and followed to adulthood in the US and in Toronto, Canada [46]. Similarly, a prospective birth cohort study of more than 1,000 children from New Zealand found no effect of BMI on AHR [23]. Other studies suggested increased AHR to methacholine and worse exercise-induced bronchospasm in obese versus non-obese children and adolescents [47-50].

### Inflammation

Adipose tissue is involved in the production and release of hormones as well as cytokines, which may both contribute to obesity-related insulin resistance, cardiovascular morbidities, hypertension, and metabolic disorders, as well as asthma [51,52]. Obesity is associated with increased adipocyte-driven pro-inflammatory activity resulting in chronic local and systemic inflammation [53,54]. One of the adipokines released by adipocytes is leptin, and serum leptin concentrations are increased in obesity. In children, increased serum leptin is associated with severity of exercise-induced bronchoconstriction [50], low-peak expiratory flow rate [55], and higher asthma prevalence in prepubertal boys [56], as well as peri- and postpubertal girls [57]. In contrast, adiponectin, which is also secreted from adipose tissue, is thought to inhibit the production and effects of pro-inflammatory cytokines such as tumor necrosis factor-α and interleukin (IL)-6 and induces the expression of the anti-inflammatory cytokines IL-1 receptor antagonist and IL-10 [52]. Serum adiponectin levels have been reported to be associated with asthma severity [33], and adverse clinical outcomes of asthma [58], as well as exercise-induced bronchoconstriction in children [50,52].

Adipokines may change the T helper cell balance in favor of a T helper cell type 1 (Th1) response, as it is seen in asthma associated with obesity [59,60]. Inflammation in allergic disease is usually dominated by Th2. However, airway disease in obese asthmatics is not typically characterized by eosinophilic/TH2 inflammation. In fact, obese children with asthma were found to have significantly higher Th1 and lower Th2 responses to specific stimuli compared with non-obese asthmatic children [61]. In support of this, studies in asthmatics have shown that increased BMI was inversely related to sputum eosinophils and FeNO [25,62,63]. Interestingly, neutrophil-predominant airway inflammation has also been described in obese women with asthma [64,65], and weight loss by exercise or dietary changes results in a decrease in airway neutrophils [66].

### Nitric oxide and ADMA

Asymmetric dimethylarginine (ADMA), a product of protein degradation, acts as competitive nitric oxide synthase (NOS) inhibitor and as such is involved in the pathogenesis of cardiovascular disease, diabetes, and asthma [67-70]. ADMA levels were increased in mouse lungs from a model of allergic airway inflammation [69] and also increased significantly in sputum from adult subjects with mild, eosinophilic asthma following a controlled inhaled allergen challenge [69]. In pediatric asthma, ADMA was found to be increased in exhaled breath condensate [71] and in sputum [69]. Sputum ADMA and decreased L-arginine:ADMA ratio, which can be used as a marker of NOS impairment, correlated with reduced FeNO [69]. The previously mentioned inverse association between BMI and FeNO in asthma [62,63] may therefore be explained by an imbalance between NOS substrate L-arginine and its inhibitor, ADMA.

Interestingly, in a cross-sectional study of participants from the Severe Asthma Research Program (SARP), subjects with late-onset asthma (>12 years) had higher ADMA and lower L-arginine plasma levels compared to subjects with early-onset asthma [72]. The L-arginine:ADMA ratio in plasma was inversely correlated with BMI in late-onset, but not early-onset asthma, suggestive of NOS impairment in obese late-onset asthma. In addition, a reduced L-arginine:ADMA ratio in late-onset asthma was also associated with less serum IgE, increased respiratory symptoms, lower lung volumes, and worse asthma quality of life [72]. These studies demonstrated that clinical-biological phenotyping may help unravel relevant pathways in asthma beyond inflammation [73].

### Nutrition and developmental programming

Certain nutrients, such as antioxidants and saturated fatty acids may represent a link between obesity and asthma [14], as they play an important role in oxidative lung damage and decreased lung defense against environmental hazards [41]. There is also increasing evidence that nutrition in infancy and even *in utero* has a major influence on later predisposition to cardiovascular, metabolic, and allergic diseases [74,75]. As recently reviewed elsewhere [41], abnormalities in the lipid and/or glucose metabolism early in life have the potential to contribute to the pathogenesis of lung disease and asthma later in life [41]. A healthy diet in pregnancy is important for lung development and innervation in the offspring, and imbalances can lead to airway hyperreactivity independent of the postnatal diet. In addition, the development of innate and adaptive immunity may also be affected by poor prenatal diet, which may result in increased susceptibility to infections with respiratory viruses such as respiratory syncytial virus and human rhinovirus, which both predispose to recurrent wheezing and asthma in childhood [41,76,77]. Metabolic and immune programming are also under epigenetic regulation, and there is increasing evidence now that nutrition can influence long-term health outcomes by modulation of epigenetic programming [78]. Interestingly, experimental studies have shown that immune programming can be modulated by the gut microbiome and that manipulation of the microbiome can prevent not only allergic disease [79,80] but also the risk of obesity, cardiovascular, and metabolic disease [81].

## Conclusions

In summary, the link between obesity and diabetes with asthma is complex. Metabolic disorders may affect the lungs in many ways, which includes changes in the mechanics of breathing, by causing or contributing to inflammation and abnormalities in the L-arginine metabolism and by modifying the metabolic and immune programming. Modification of the epigenetic programming through intrauterine exposures to environmental triggers including altered lipid and/or glucose metabolism of the mother may contribute to the observed increase in incident pediatric asthma. Supportive interventions in the treatment of obese/overweight asthmatics should include weight loss.

## Abbreviations

ADMA: asymmetric dimethylarginine
AHR: airway hyperresponsiveness
BMI: body mass index
CAMP: Childhood Asthma Management Program
FeNO: fraction of exhaled nitric oxide
IgE: immunoglobulin E
IL: interleukin
NOS: nitric oxide synthase
Th: T helper cell
